# Experience reports from active volunteer members in German right-to-die organisations through qualitative content analysis

**DOI:** 10.1186/s12910-025-01263-9

**Published:** 2025-07-17

**Authors:** Sandy Ohm, Claudia Bozzaro

**Affiliations:** 1 Faculty of Medicine, CAU Kiel University, Kiel, Germany; 2 Department of Medical Ethics, University of Münster, Münster, Germany

**Keywords:** Right-to-die organisations, Assisted suicide, Assisted dying, Volunteer work, Experience reports

## Abstract

**Background:**

Although not widely known to the general public, right-to-die organisations have been active in Germany since the 1980s, similar to Switzerland. Notably, there is often a lack of focus on the volunteer members of these organisations, despite their unique perspectives on the matter. Our study aims to thoroughly investigate the experiences of members in German right-to-die organisations and thereby make a significant contribution to the ongoing debate.

**Methods:**

This project was implemented through qualitative interviews conducted with active volunteer members of German right-to-die organisations, followed by a qualitative structured content analysis based on Mayring’s approach. Using a coding evaluation method, relevant aspects were extracted from the data and on one hand assigned to predetermined categories, while one the other hand, new categories emerged organically from the material.

**Results:**

After conducting 13 interviews, it became clear that, while some participants were deeply impacted by the dramatic nature of the illness-related circumstances surrounding certain assisted suicides, the majority do not find the work burdensome. Instead, they view it as highly fulfilling and rewarding. In particular, interacting with the relatives of those seeking assistance and with the individuals themselves is seen as especially enriching. Furthermore, participants frequently encounter significant ignorance from large segments of the medical community and law enforcement regarding current regulations, which can lead to complications during assisted suicides. Additionally, collaboration with care facilities and senior residences presents a considerable challenge for many members, often due to religious objections.

**Conclusion:**

Our study of volunteer members of right-to-die organisations is, as far as we know, the first one with active volunteers of German right-to-die organisations. The interviews thus provide new and important insights into a lived practice that has been little studied. These insights must be incorporated into the current debate on the responsible establishment of the practice of assisted suicide.

**Supplementary Information:**

The online version contains supplementary material available at 10.1186/s12910-025-01263-9.

## Background

In Germany the question of how to handle suicide assistance has been and still is the focus of intense debates in the socio-political as well as in the scientific context. For a long time, assisting suicide was not regulated by law in Germany, whereas voluntary euthanasia has been legally prohibited since the introduction of the Criminal Code in 1871 under Sect. 216.

German-speaking countries, in particular, struggle with the use of the term ‘euthanasia’ due to its association with the Nazi regime’s mass murder programs in 20th-century Germany. Instead, alternative terms such as ‘active assisted dying’ or ‘killing on request’ are preferred [15].

Nevertheless, the topic remained taboo both socially and within the medical profession. One important reason was that the German Medical Association defined assistance in suicide as a non-medical practice, which implied that physicians performing assisted suicide could be potentially sanctioned by losing their approbation (right to work). As a result, the first German right-to-die organisation, the *Deutsche Gesellschaft für Humanes Sterben* (DGHS), was founded in 1980 with the aim of strengthening the right to a self-determined death and, above all, offering a means of assisted suicide [8].

As right-to-die organisations became more prevalent, parliamentarians made their first attempts to establish legal regulations in 2012. This eventually led to the German Bundestag officially criminalising suicide assistance in December 2015 through Sect. 217 of the Criminal Code, which effectively made it almost impossible for German right-to-die organisations to operate [16]. But after lawsuits filed by doctors and members of assisted dying associations the Federal Constitutional Court declared the law introduced in 2015 as unconstitutional and consequently overturned the section in February 2020 [14]. As a result of this legal change, the German Medical Association was also compelled to amend the Professional Code for Physicians, because until then, Paragraph 16 had prohibited physicians from providing assistance with suicide.

It has now been five years since the repeal of Sect. 217, and after the German Bundestag rejected two bills from cross-party groups in July 2023 to re-regulate assisted suicide [17] there is still no law governing assisted suicide in the future. This situation benefits German right-to-die organisations, which had previously been restricted by the former regulations but now enjoy considerable freedom in their work. But although public discourse frequently discusses organisations supporting assisted dying, the public knows relatively little about their actual work. This raises the question of who the people are that choose to volunteer in this field and what experiences they have had in the context of assisting suicides. After all, these are the very people whose work has been the subject of much media attention for several years now, as demonstrated by various news articles published over the years [36, 37]. However, some of the organisations are not entirely comfortable with this publicity and are reluctant to provide deeper insights, as our attempts to contact the organisations during the course of the research will show.

### Overview of German right-to-die organisations

In Germany, three associations are primarily relevant in the context of assisted suicide: the *Deutsche Gesellschaft für Humanes Sterben*, abbreviated DGHS (https://www.dghs.de), *Dignitas* (https://www.dignitas.de/) and the *Verein Sterbehilfe* (https://www.sterbehilfe.de).

The *Deutsche Gesellschaft für Humanes Sterben* is an organisation that has been active for over 40 years and was founded in 1980 in Nuremberg. According to its own statements, it was the first German organisation to play a leading role in the development and promotion of the living will in Germany [8]. The *Dignitas* association originated in Switzerland and was founded in 1998 and later on established its German branch in 2006 [11].

Additionally, the *Verein Sterbehilfe*, based in Hamburg, is the youngest of the three organisations and was founded in 2009 [38].

It should be noted that not every association refers to itself as an “assisted dying organisation” or “right-to-die organisation”. For example, the *DGHS* describes itself as a “civil rights and patient protection organisation,” emphasizing its broader scope of activities. This includes, for instance, the development and execution of advance healthcare directives and powers of attorney, offering end-of-life counselling or organising regular discussion groups [9]. *Dignitas* and the *Verein Sterbehilfe* also support their members in developing advance directives and offer advisory services [12, 39].

To enhance transparency regarding the number of assisted suicides, the *DGHS* and *Verein Sterbehilfe* publish annual press releases with current case numbers. According to these reports, *DGHS* facilitated a total of 623 assisted suicides in 2024, while *Verein Sterbehilfe* accompanied 171 cases [10, 38]. Official figures from *Dignitas* are not available, but according to an article on the *Rheinische Post* website, the organisation carried out a total of 183 assisted suicides in 2024 [28].

### Aim of the study

The literature on physician-assisted suicide approaches this topic from different perspectives: there are studies, for instance, on the attitudes of the medical profession toward assisted suicide [20, 24, 30], as well as articles that address the fundamental ethical debate [18, 32, 35]. There are also occasional studies on euthanasia organisations, which mostly focus on cases involving the Swiss organisations EXIT or Dignitas. In these retrospective analyses, all assisted suicides between 1990 and 2000 were examined, with a focus on the underlying medical conditions and sociodemographic characteristics [3]. In another study, all cases from the organisations Exit Deutsche Schweiz and Dignitas between 2001 and 2004 were analysed and compared with previous cases, also in terms of medical conditions and sociodemographic characteristics [19].

However, it is clear that despite intensive research, there is relatively little literature that addresses the experiences of volunteer members of such organisations. Given the political relevance, this further highlights the importance of conducting studies on members of right-to-die organisations, especially in German-speaking countries. These studies could provide valuable insights in the societal debate and offer a different perspective on the issue.

The aim of this paper is, therefore, to provide a detailed examination of personal experiences of volunteer members of German right-to-die organisations and provide a first insight into this group’s perspectives.

## Methods

In this study, qualitative interviews were conducted with volunteer members of one of three major German right-to-die organisations. In these expert interviews, the respondents share their personal experiences and perspectives, offering insights that help to answer the research question. The semi-structured interviews follow a question guide that we specifically developed for this purpose, where the interviewees are asked to respond to open-ended questions in a flexible and open conversation [27].

The interviews were recorded and subsequently transcribed with the analysis software MAXQDA. During transcription, personal data were anonymised, and each interview partner was assigned an abbreviation (I1, I2,. up to I13) as part of the pseudonymisation process. Following this, a qualitative structured content analysis according to Mayring [23] was conducted. The method of qualitative structured content analysis according to Mayring describes a coding evaluation procedure aimed at extracting specific aspects from the data and categorizing the material according to predetermined organizational criteria.

### Recruitment of participants

Before presenting the results, the recruitment of interview participants should be mentioned in more detail. Initially, it seemed challenging to establish contact with members within the organisations, as the first contact with the responsible individuals showed little to no interest in forwarding the request internally. However, considering the sensitive nature of the topic and the desire to protect members from seemingly insincere inquiries, this behaviour is somewhat understandable. Attending a member meeting of one of the organisations led to some productive conversations, and additional interested individuals were recruited through flyers. The proactive phone contact with representatives across Germany, whose numbers are publicly available on the organisation’s website for inquiries and consultations, was also successful. Ultimately, despite repeated attempts to establish contact with all three right-to-die organisations, only members from one organisation were willing to speak with us. Due to the anonymisation of the participants, the organisation will not be named.

## Results

After recruiting the participants, a total of 13 interviews were conducted between July and October 2023. The decision to conclude data collection at this point was based on data saturation. It became evident that no fundamentally new aspects emerged in later interviews and the key themes had been sufficiently explored. Since the collected data already provided rich and meaningful insights, further interviews would not have significantly enhanced the study’s findings. After transcribing the audio files and conducting a structured qualitative content analysis according to Mayring, a total of 13 categories were created.

**Table 1:** Overview of abbreviations, role in organisation and length of the interview

abbrevations	role in organisation	length of interview
I1	contact person	31 min
I2	contact person	1 h, 8 min
I3	contact person	32 min
I4	board member	47 min
I5	contact person	36 min
I6	contact person	32 min
I7	board member	1 h, 2 min
I8	physician	1 h, 55 min
I9	physician	1 h, 3 min
I10	physician	33 min
I11	contact person	55 min
I12	physician	51 min
I13	physician	48 min

For a better context of the results, it is useful to describe the participants and their biographical characteristics. After completing the interviews, it became clear that the gender distribution, with seven women and six men, is quite balanced. Furthermore, the age distribution shows that three participants are under 60 years old, nine participants are between 60 and 79 years old, and one person is over 80. Thus, the majority of participants are already of retirement age or are close to it. Although age and the previous profession do not play a central role in the interviews, it is worthwhile to briefly mention them. Specific occupations cannot be named due to anonymisation, but it can generally be said that most participants had academic backgrounds, as seen in Table 1.

The few studies available on members of right-to-die organisations reveal that members are most commonly over 65 years old [7] and almost 90% are over 55 years old [21]. Additionally, members tend to have a higher socioeconomic status and education level [7, 40].

The duration of membership in the organisation was also discussed in the interviews, with different time periods being mentioned. The majority of participants had been active for many years at the time of the survey, with most time frames ranging between 10 and 20 years. Two participants even indicated that they had been active in the organisation for 40 years. Notably, the doctors often reported shorter engagements, ranging from 2 to 4 years, which is likely due to the legal change in 2020 that has once again allowed them to legally assist with suicide.

**Table 2:** Overview of the category system

motivations	experiences	expectations
negative encounters with end-of-life circumstances	memorable cases	opinions on the political situation
altruistic factors	role of medical profession	desired future approach to assisted suicide in Germany
medical motivation	challenges and obstacles in assistance	death culture
suicide prevention	psychological effect of organisation work	
	engaging with relatives	
	religious aspects	

The category system in our study was divided into three thematic sections: motivations, experiences, and expectations. Each section consists of content-related categories that contribute to answering the research question, as Table 2 shows. This paper focuses only on the “experiences” section, as the other categories are covered in separate publications.

For the purposes of this paper, the categories are further structured according to positive and negative aspects of organisational work and will be discussed in detail in the following sections, beginning with the positive more enriching experiences and concluding with the more burdensome and negative aspects as described by the participants.

### Positive aspects of the organisation’s work

#### Engaging with relatives

A point frequently raised by participants was the importance and sometimes difficulty in dealing with family members. Often, those wishing to die hesitate to discuss their intentions with their families due to fear of rejection. Others are concerned that the emotionally taxing situation makes it harder for family members and friends to accept and therefore carry out the plan. This can become particularly problematic for the volunteers when it comes to deciding on the location for the planned suicide, for instance, if family members do not want the assistance to take place in the shared home. An example illustrating how a rejecting family member can interfere with the work of suicide companions is provided by participant I12:

“This man had severe lung cancer with multiple metastases, but the wife was strictly against it. She wanted her husband to continue living or to die a natural death and was even willing to tear out the infusion line during the assistance.” (I12).

Participant I7 further elaborates on the difficulty of accepting the wish of a loved one to die. According to her observation, the children of individuals considering assisted suicide often struggle more with letting go of their parents and supporting them in this process than their spouses do. The spouses are often much more directly involved and can better understand the suffering their partner is going through.

“And a child, as I interpret it, […] always experiences that it was the very purpose of its parents’ lives, especially the mother’s. A child is helpless but senses that it holds enormous power—the power to give its mother a sense of purpose in life. […]. And then, at some point, this mother says, ‘I don’t want to live anymore […]. This is a blow to one’s sense of self, a kind of affront, and that’s why it manifests in statements like, ‘Oh no, you must not choose assisted suicide.‘” (I7).

The interviews revealed positive experiences with family members as well, and the participants emphasized how enriching and valuable the experience can be for both the person wishing to die and their loved ones. While family members often struggle initially with the idea of accompanying the dying person through assisted suicide, these final moments are particularly crucial for those left behind to process their grief, as participant I8 explains.

“The people who stay behind, they need this feeling—something I am fortunate enough to witness every time—this harmony, this peace, this truly incredible spirit. They actually need it to come to terms with the situation.” (I8).

Participants also reported that the moments before administering the life-ending medication are often used by families to spend meaningful time together and celebrate the person’s life.

“It was summer, in the morning. The car pulled up, and five people got out—the woman, her husband, two sons, and a daughter-in-law. They took out two small bottles of sparkling wine. They stood outside for another half an hour, maybe 45 minutes, drinking together, and then the woman said: ‘I think, my dear ones, now the time has come.‘” (I2).

“And often, it creates this beautiful setting when family members are there, when children are present. Or when, for example, the spouse holds the person wishing to die in their arms, or in whatever way feels right.” (I13).

### Psychological effect of organisation work

Given the nature of this work, it is inevitable that questions arise about the mental effects that come with engaging in such a role. Participant I1 confirms that one must develop a “thick skin” for this work, and others mention that they are grateful to be able to carry out this work and are able to cope well with the fates they are confronted with.

“And it may be wrong to say, but I am proud that I can do this, that I can handle it […], that I am able to do this. Because I know that there are very few people who can do this.” (I8).

In fact, some volunteers also describe situations in which they struggled more with the psychological burdens of this work and felt the impact on their mental health more acutely. For example, I2 finds it challenging to meet with strangers regularly and emotionally adapt to these individuals each time. However, regular conversations with his wife and occasionally a glass of wine help him cope with such situations better.

“Well, this is a heavy burden, but of course also the responsibility you take on as an end-of-life helper, and also the […] responsibility to get everything right.” (I4).

On the other hand, the majority of participants reported positive mental effects from this work, regularly highlighting in the interviews how fulfilling they found it. In particular, the gratitude expressed by those wishing to die is described as unique in this context and difficult to compare with other kinds of work.

“…what I experience is an incredible gratitude from the people. […] So when they really thank you before they start the process, thanking you for making this possible […]. I’ve never received as much gratitude from my living patients as I have from those who have died.” (I10).

But it is not only the expressed gratitude that is frequently mentioned as a factor, but also the actual interaction with the individuals wishing to die that stands out as particularly enriching for many participants. Some participants share stories of deep friendships they have formed with people they met through their work with those seeking assisted suicide.

“[…] there are always people with whom you form a special bond. It’s just like that, the last suicide assistance I was part of, it was just a deep friendship, even though we had only known each other for six months.” (I3).

Occasionally, however, it became apparent that some participants spoke with exceptional enthusiasm and almost euphoria about the process of accompaniment, raising the question of whether this work might play an identity-shaping role and, over time, could potentially become a self-serving purpose.

“Yes, last week I had seven suicides. […] And there is no one who has done more assistances than I have. I am now at about 260 in two and a half years, and it’s increasing daily.” (I12).

“I also feel that I’m no longer just a persona there, but I am available […] in accompanying them to the threshold; they must then take the leap themselves. So, like a travel companion into the unknown…” (I9).

“And it was pure bliss. It was so quiet, such a very beautiful, calm, I don’t even know how to describe this atmosphere. It might have been something heavenly.” (I3).

### Negative aspects of the organisation’s work

#### Memorable cases

During the interviews, participants frequently recounted past suicide assistances that had left a lasting impression on them due to the dramatic circumstances of the underlying condition. For example, some participants shared cases involving severe cancers, such as individuals with breast cancer or skin cancer that led to dramatic metastases.

“This woman in her early 60s who had a very advanced cancer, a specific type of breast-cancer. […] However, she had a unique form of metastasis—namely, exclusively cutaneous metastases. These grew around the entire circumference of her thorax, forming fist-sized cauliflower-like growths, and this had been going on for three or four years.” (I8).

Another participant described a case involving a 45-year-old woman who, as a child, had been sold by her parents to pedophiles, an experience that left a profound impression on him.

“During the initial consultation the psychiatrist who had known her for 20 years was present. He said to me, ‘This woman hasn’t had a single happy day in her life.’ She even changed her name so that the family who abused her couldn’t find her anymore… And then she said to me as her last sentence before she fell asleep, ‘Now I’m finally going home.’ That hits you right in the heart.” (I12)​

#### Role of medical profession

Throughout the interviews, participants reported that doctors are often uninformed and still believe that assisted suicide is illegal in Germany, despite the extensive media coverage in recent years. Furthermore, some of the participating doctors reported that they often notice that many physicians, due to a lack of knowledge about the legal situation, are concerned about legal consequences and are thus rather averse to the topic.

“They say, “That’s not possible here in Germany” and even though there are articles about it in newspapers, in daily publications, and in the German Medical Journal. I don’t know what my colleagues are reading.” (I13)

“I believe most (physicians) are of course afraid of potential legal consequences because they are not fully familiar with the situation.” (I8)

Two participants experienced that doctors justified their reticence by citing the Hippocratic Oath, arguing that they had sworn it. This particularly irritated participant I9, as he, as a physician, knew firsthand that no doctor living today is required to swear an oath.

“I mean, the whole Hippocratic Oath thing—some doctors have also thrown that in my face. […] And I don’t know any living doctor who has actually sworn any kind of oath. But even things like that were completely far-fetched, just to, how should I put it, have something to hide behind.” (I9)

Other participants reported specific negative attitudes in their collaboration with doctors, often with general practitioners. Several mentioned that they often hear from those wishing to die that they cannot expect support from their general practitioner, as the latter has clearly spoken out against it. Others also reported in the interviews that they had already asked their personal general practitioners to collaborate with the right-to-die organisation, but they also encountered rejection there.

“I keep hearing that people go to their general practitioner, and the doctor says, ‘If you bring up this topic again, I’ll send you to psychiatry.‘” (I10)

A frequently mentioned point addresses the fundamental principles of medical practice, specifically the task of healing. Several participants view this as one of the reasons for the common rejection of assisted suicide. Some of the participants who are physicians themself further explain that doctors are trained from the beginning of their careers to focus primarily on healing and alleviating symptoms, always keeping another therapeutic method in mind that can be offered to the patient. Therefore, the idea of assisting a patient in an intentional suicide is difficult for many doctors to comprehend.

“[…] Doctors feel like failures in that moment, and they don’t want to appear as such, but in my opinion, that’s a misguided sense of ambition. […] Admitting that they couldn’t help in that case seems to be something doctors have a problem with.” (I8)

Participants also described that the general approach to death is not as simple for many doctors as one might expect from this profession. Participant I8, who is a doctor herself, shares her experience that certain topics are often avoided by medical colleagues.

“So, I’ve already noticed, even independently from these assisted suicide cases, that many doctors have no interest in dealing with the topics of death, dying, therapy limitations, or conversations with family members. […] Many doctors prefer to remain very factual. And because, in a way, they can protect themselves from emotionality and distance themselves.” (I8)

#### Challenges and obstacle in assistance

In discussions about past assisted suicide cases, participants highlighted recurring aspects of the work that they found particularly challenging or problematic. These areas appear to regularly give rise to uncertainties, ethical dilemmas, or organisational difficulties in day-to-day practice. The following section provides a closer examination of these key issues, illustrated with selected interview excerpts.

##### Criminal Police or public prosecutors

When reflecting on the difficulties the participants had encountered, a recurring pattern became apparent: They reported problems in collaborating with law enforcement agencies such as the criminal police or public prosecutors. One particular point of importance, which was already discussed in the previous section, is a significant lack of knowledge. The volunteers encounter police officers and even judicial authorities who are not yet aware of the ruling by the Federal Constitutional Court. To avoid potential discussions, I2 describes that he always keeps a written copy of the ruling ready to show the officers, fully aware that it facilitates a quicker handling of such situations.

“I was once in the area near (region) […]. When the criminal investigator came he said, ‘Here in (city), what the Federal Constitutional Court in Karlsruhe decides does not apply here in (city).‘” (I12)

Participants described that this lack of relevant information about the current legal situation repeatedly leads to stressful and avoidable situations at the deathbed of the individuals seeking to die. Several participants recount uncomfortable moments when the police stormed the location of death with flashing lights and a large response team after the suicide was properly reported by the assisted dying companions. For instance, I9 reports about the first accompaniment he conducted, during which several police officers entered the apartment, and shortly thereafter, a large emergency medical team also arrived. Another participant reports a similar experience:

“And then I called the criminal police and said, ‘Here, I have a medically assisted suicide with a woman in such-and-such street. Please send the criminal police.’ Nobody came. After an hour, I called again. They thought it was a prank call because this doesn’t exist in Germany, they told me. [.] Then they arrived an hour later, three officers in bulletproof vests. Each of them had two pistols, one on each side. And they stood there in front of a 90-year-old friend of the deceased.” (I12)

##### Experiences with care facilities

The topic of assisted suicide in healthcare extends beyond just the medical profession, as institutions such as care facilities and senior residences are increasingly confronted with requests for assisted dying. In conversations with volunteers, nursing homes were often a topic of discussion, as those wishing to die frequently express that they do not want to go into a home under any circumstances.

“That’s one of the most common arguments I hear. ‘I definitely won’t go to a nursing home.’ […] Many of my patients are familiar with nursing homes, whether because they had relatives there or because they themselves were temporarily placed in one. And they say, ‘No, I’m not putting myself through that again.’ (I13)

However, care facilities were also mentioned in the interviews in a different context, namely due to significant problems in collaboration. Volunteers report that they are often quickly blocked by the management and are even expelled from the premises when consulting and visiting individuals that reside in care facilities. Participant I12 reports that he has been banned from several facilities and that, particularly during one accompaniment, the management threatened him with legal consequences. The Participants I4 and I11 describe that many institutions fear suggesting a false image to the residents, thereby potentially encouraging them towards suicide, which could ultimately harm the institution’s reputation and image.

“I had conversations with other nursing homes, and they say, ‘Yes, but we’re afraid that the next ones will say, ‘Oh, then I want to leave too,’ and the others will say, ‘What kind of a place is this? I don’t want to be here where people are being killed,’ just to put it bluntly.” (I11)

I3 elaborates on this argument and suspects that these institutions do not act out of callousness but rather see economic pressure as a primary concern.

“… then I get sent home by the management because it’s a Catholic home. And then you’re just left standing there, and they won’t allow it to be done in their home. And then we have to do these little tricks […], like taking the lady or the man out of the home briefly, and then going to the funeral director, so that they can die there.” (I3)

#### Religious aspects

Building upon the previous section on care facilities, it is thematically appropriate to take a closer look to the religious experiences associated with the work in right-to-die organisations. In fact, many of the previously reported experiences took place in religious care facilities, specifically Catholic ones.

Another point discussed in the religious context is that some volunteers have faced criticism for their involvement in a right-to-die organisation from religious individuals. For instance, two participants reported that they were verbally attacked and judged for their involvement with the organisations.

“And then a lady started to insult me, sitting in a wheelchair, saying, ‘Do you actually know that what you’re doing is a mortal sin? You’re inciting murder. You’ll burn in hell.‘” (I2)

“Where I faced more opposition was from church representatives. […] And at one point, I was even labeled a potential murderer for advocating it.” (I7)

While participant I6 did not encounter such dramatic confrontations with religious individuals, she related to the taboo surrounding discussions of suicide and death within the Christian community. She lives in an area where Christianity is still deeply rooted and often finds that she cannot talk about death with her neighbours or acquaintances without someone dismissing the topic in absolute reverence, claiming that even speaking of it is a sin.

It is not only the staunch opponents of assisted suicide who are caught in a religious dilemma, but also those directly involved in the process. According to one participant, it is not uncommon for individuals seeking assisted suicide to be torn between their spiritual beliefs and their desire to end their lives.

“And it is also difficult for people who believe, who fear damnation because of it. […] And then I always say, ‘We weren’t asked if we wanted to come into the world. So, in the end, you could turn it around and say, then I don’t want to know who could forbid me to do that.’ And then you can already see that people are relieved to be able to say, ‘Yes, the damnation won’t come.’ (I3)

## Discussion

At the beginning of the paper, the unique aspects of interacting with family members were discussed and a certain reluctance was noted among family members who initially have concerns about accompanying their loved one through assisted suicide. Likewise, those affected tend to be cautious when expressing their own wish to die, fearing rejection.

In fact, the results of a study on 114 approved requests for assisted suicide through the organization Exit in Switzerland show that in 75% of cases, family members supported the decision of the affected person, while only in 5% of cases, relatives were unanimously opposed [4]. This suggests that in most cases it is possible to involve family members in the process, enriching the experience for both the individual and their loved ones.

Regarding the psychological effects of suicide assistance, studies primarily focus on its impact on the medical profession. These often suggest that involvement in the process can have significant emotional effects and that many doctors would generally prefer to avoid such a difficult and absorbing situation [2, 26, 29]. A 2011 survey of 1,456 Dutch physicians also reveals that 86% expressed concerns about the emotional burden of personal involvement [13]. In our study, although some participants describe similar experiences of emotional strain, the overarching message is that this work is perceived as highly fulfilling and enriching overall.

In the section on past suicide assistances, the aspect of the underlying illness was often a focal point, a topic that is also widely discussed in the societal context. While severe malignant diseases, such as the cancers described earlier, do frequently appear in the context of assisted suicides, they do not constitute the majority of the motivations for those seeking assistance. A look at the case numbers from DGHS, the largest German right-to-die organisation, shows that in 2024, of the 623 assisted suicides conducted, only 21.83% cited cancer as the main reason. The most common motivation, however, was multimorbidity, with 27.77%, followed by tiredness of life with 22.15% [10].

Furthermore, literature suggests that psychosocial factors play a larger role in motivation than might initially be assumed. For example, the loss of autonomy and dignity, increasing dependency, and diminished quality of life are often cited as the main reasons [5, 13, 25]. Additional studies also show that the image of a severely ill and pain-ridden person does not always reflect the reality, and physical pain is not necessarily the primary motivation for seeking assisted suicide [6, 41].

The detailed accounts from the volunteers also show that the medical profession often occupies a special position in the discussion surrounding assisted suicide. This is likely due to the fact that, especially in end-of-life matters, physicians play an essential role as caregivers and are particularly important in the context of assisted suicide. They are crucial for obtaining the life-ending medication and are often responsible for assessing the decision-making capacity of individuals seeking assistance to die. The lack of knowledge and lack of cooperation our participants observed therefore reveal a significant problem in the daily work of the volunteers.

However, this lack of knowledge is not a new finding, as a study on German medical students from 2015 shows. Shortly before the introduction of Paragraph 217 the students were asked about the legal situations on different cases surrounding end of life choices and it showed that while 93.7% of students were correctly informed about the prohibition of euthanasia and 81.2% about the legality of palliative sedation, only 18.8% knew about the legal immunity associated with physician-assisted suicide [1].

The observed reluctance, especially from general practitioners, is surprising, as the existing literature shows that general practitioners are more likely to be supportive of assisted suicide and more willing to approve it [2, 26]. This could be due to the close and often long-standing relationships that general practitioners tend to have with their patients, which might result in a greater understanding of individual suffering [2, 34].

However, the question arises as to whether this special position is justified and whether assisted suicide should be an integral part of medical practice. Many physicians find it challenging, particularly when it comes to individuals who are not primarily suffering from physical ailments, to make decisions about their death. They argue that such decisions should not fall within the medical domain [33].

Finally, religious aspects on the part of relatives, care institutions, or churches were also a relevant point for the participants. The fact that religious objections play a significant role in the opposition to euthanasia and assisted suicide is not a recent discovery. Numerous population studies show that religious affiliation significantly impacts views on issues such as euthanasia, suicide, and abortion, often leading to a more negative stance [31, 35].

Conversely, individuals without religious affiliation tend to have a more open stance on topics related to euthanasia [13]. However, it should be emphasized that there are also differences in opinion within various religious denominations. One study reveals that, with regard to euthanasia, Protestants tend to be more supportive than Catholics, and Catholics, in turn, more supportive than Muslims [24]. The influence of religion on personal views also extends to the medical profession. Numerous studies show that religious physicians are more likely to reject requests for assisted suicide than their non-religious counterparts [20, 22, 23].

A study from 2020 provides an additional important aspect: The article highlights that in Christianity and Judaism, suicide is seen as a grave religious transgression, with significant punishments in the afterlife. As a result, participating in assisting a suicide is strongly feared by religious individuals due to the potential consequences in the afterlife. An interesting point raised in this context was that belief in the existence of Hell has a stronger influence on negative attitudes toward assisted suicide than belief in Heaven. The author concludes that fear of eternal punishment has a particularly strong influence on opinions [31].

The sum of these insights has not been sufficiently documented in the broader literature and once again highlights the need to address these aspects more prominently in the public debate, particularly in the scientific context.

### Limitations

When critically reviewing the study, a few limitations can be identified, which should be mentioned to improve transparency and contextualize the results. First, due to the relatively small sample size of 13 participants, the study does not represent the entirety of volunteers within German right-to-die organisations. Considering the core aspects of qualitative social research, however, this study is less focused on statistical representativity and more on capturing detailed individual perspectives. As such, the emphasis is on the depth of insights rather than the scale of the sample.

Additionally, it should be once more noted that the majority of participants are active in the same organisation, which could potentially lead to similar experiences, even though each participant naturally processes and perceives these experiences individually. Finally, in the interest of scientific neutrality, it must be acknowledged that a certain degree of personal bias cannot be entirely avoided. Especially in the context of the interviews, a certain level of suggestion through the open conversation format with participants can hardly be fully excluded.

## Conclusions

Our study reveals that participants acknowledge the mental challenges of this role, especially regarding the dramatic nature of some assistances, but overall report positive psychological effects, highlighting the fulfilment of this work. In particular, the gratitude of the individuals concerned and deep emotional connections with those seeking assisted suicide were mentioned. The challenges of involving family members in assisted suicide were also of importance, with many individuals hesitating to share their wishes with family due to fear of rejection. However, despite initial struggles, the final moments were often viewed as valuable for both the dying person and their loved ones. A widespread lack of knowledge among many medical professionals and law enforcement agencies, such as the police and public prosecutors, regarding current legal regulations frequently leads to complications during assisted suicide cases. Additionally, collaboration with care facilities and senior residences also poses significant challenges in the practice of assisted dying, often due to religious beliefs and values that can influence attitudes towards assisted suicide.

Given the limited research on the experiences of volunteers in right-to-die organisations, this work makes an important contribution to advancing the understanding of the subject. It is, as far as we know, the first study with active volunteers of German right-to-die organisations and not only provides valuable insights into the individual experiences of the volunteers but also introduces perspectives that have been previously overlooked in academic discussions. As such, this research significantly enriches the current field and opens new avenues for future studies, as well as deepening the understanding of volunteers’ perspectives in the context of assisted suicide. To advance the overall debate, it would be beneficial to address these challenges in more detail, in order to identify areas that require more action and that may hinder the practical application of theoretical concepts.

## Electronic supplementary material

Below is the link to the electronic supplementary material

Supplementary Material 1

## Data Availability

The data that support the findings of this study consist of interview transcripts and are not openly available due to their sensitive nature. To protect the anonymity and confidentiality of the participants, the data can only be made available upon reasonable request from the corresponding author.
