# Supplementary material for: Experience reports from active volunteer members in German right-to-die organisations through qualitative content analysis
Source: BMC Med Ethics. 2025 Jul 17;26:98. doi: 10.1186/s12910-025-01263-9 (PMC12273378; doi:10.1186/s12910-025-01263-9)
Supplement: Supplementary file 1 — Supplementary Material 1 [file 12910_2025_1263_MOESM1_ESM.pdf]

Interview guide for study on volunteer members in German right-to-die organisations

Sandy Ohm, Claudia Bozzaro

1.) How long have you been involved in the organisation, and what are your responsibilities?

2.) What initially motivated you to become a member?
(Has this motivation changed over time?)

3.) How would you describe the meaning or purpose of your work within the organisation?

4.) What kind of key experiences have you had in this work so far?
(Moments that left an impression)

5.) What experiences have you had in working with physicians?

6.) Have you ever encountered personal limits in the course of this work?
(Does the work affect you mentally?)

7.) What would your ideal vision for future legislation look like?

8.) How would you envision an optimal approach to assisted suicide in Germany?
